# Pharma 4.0 Continuous mRNA Drug Products Manufacturing †

**DOI:** 10.3390/pharmaceutics13091371

**Published:** 2021-08-31

**Authors:** Andreas Ouranidis, Christina Davidopoulou, Reald-Konstantinos Tashi, Kyriakos Kachrimanis

**Affiliations:** 1 Department of Pharmaceutical Technology, School of Pharmacy, Aristotle University of Thessaloniki, 54124 Thessaloniki, Greece; cdavidof@pharm.auth.gr; 2 Department of Chemical Engineering, Aristotle University of Thessaloniki, 54124 Thessaloniki, Greece; rtasi@cheng.auth.gr

**Keywords:** systems-based, pharma 4.0, digital design, continuous mRNA manufacturing, RNA vaccines, continuous RNA vaccine manufacturing, mRNA vaccines, Covid-19, Monte-Carlo, SARS-CoV-2, Aspen mRNA process flow, lipid mRNA formulation, in silico design

## Abstract

Continuous mRNA drugs manufacturing is perceived to nurture flow processes featuring quality by design, controlled automation, real time validation, robustness, and reproducibility, pertaining to regulatory harmonization. However, the actual adaptation of the latter remains elusive, hence batch-to-continuous transition would a priori necessitate holistic process understanding. In addition, the cost related to experimental, pilot manufacturing lines development and operations thereof renders such venture prohibitive. Systems-based Pharmaceutics 4.0 digital design enabling tools, i.e., converging mass and energy balance simulations, Monte-Carlo machine learning iterations, and spatial arrangement analysis were recruited herein to overcome the aforementioned barriers. The primary objective of this work is to hierarchically design the related bioprocesses, embedded in scalable devices, compatible with continuous operation. Our secondary objective is to harvest the obtained technological data and conduct resource commitment analysis. We herein demonstrate for first time the feasibility of the continuous, end-to-end production of sterile mRNA formulated into lipid nanocarriers, defining the equipment specifications and the desired operational space. Moreover, we find that the cell lysis modules and the linearization enzymes ascend as the principal resource-intensive model factors, accounting for 40% and 42% of the equipment and raw material, respectively. We calculate MSPD 1.30–1.45 €, demonstrating low margin lifecycle fluctuation.

## 1. Introduction

Since the early 1990s, Wolff et al. [1] have demonstrated the feasibility of protein expression to rodents by injecting naked mRNA. The driving clinical force behind RNA-based drug development remained unchanged, hence the mechanism of action neither causes genomic integrational mutation nor promotes toxic immune responses [2]. In addition, due to its transient bioavailability profile, RNA administration may be discontinued and/or regulated. The modular, synthetic character of mRNA vaccine design spans the principles of pharma 4.0 paradigm, encompassing a data-intensive, universal platform, hence any gene of interest, even if exogenous, may be encoded and subsequently embodied to a purpose-specific transfection vector [3]. Moreover, the central of RNA production steps, namely the in vitro transcription (IVT) reaction, does not require viral promoter inclusion, which may be associated with dual risks of uncontrolled genomic insertions and pathogenic changes of protein expression. 

Despite the acknowledged benefits, the clinical application of mRNA remained elusive for two decades, reflecting the instability and immunogenicity-related toxicity concerns. Standing on the shoulders of giants, though, these issues have currently been addressed to satisfactory extent by methodologies described in the scientific literature, such as those developed over more than three decades by the Karikó lab [4,5,6]. As a result, the administration of RNA, encoding the genetic region of interest, currently ascends as an effective, potent, non-infective, transient, and therefore relevantly safe molecular tool, one to mechanistically exploit the translational dogma of biology towards the on demand peptidic biosynthesis, thus enabling interventions of previously unseen precision [7]. 

However, RNA-based vaccine manufacturing, as with all biopharmaceuticals repertoire, relies on batch operations which are characterized by low-level automation and reproducibility [8]. Owing to the latter, production steps must be performed by highly trained personnel in accredited facilities, which in turn concomitantly raises the standard operating procedures (SOPs) enactment difficulty, narrowing the prospects of production transfer to local settings. These manufacturing hurdles, in synergy with documented logistical barriers [9] disturb the supply chain in a pandemic crisis, an evident reality faced during the recent global Covid-19 outbreak, whereas regional facilities capable of taking-up immediate vaccine production were limited, if not nonexistent. 

Therefore, a novel, flexible, decentralized manufacturing paradigm needs to be explored, one to surpass the mentioned setbacks, defending humanity against pandemic outbreaks. Expanding the former statement, automation, reproducibility robustness, and real-time analytical validation challenges may be addressed by continuous processing [8]. Since such a good manufacturing practice (GMP) compliant facility mockup would sacrifice prohibitory resources to realize, first-principles and systems-based design utilizing available 4.0 industrial tools would provide invaluable insight to the equipment and operational specifications, thus ensuring, within given limitations, credible production planning, capacity, and cost forecast. Such a model would be capable of suggesting a baseline of exploiting existing inventories in order to respond to an urgent pandemic incident. 

The primary objective of this research is to realize this model implementation by utilizing in silico hierarchical design of the related bioprocesses, embedded in scalable devices, compatible with continuous operation. Our secondary objective is to evaluate the feasibility aspects of establishing a flexible, modular, GMP production line for the end-to-end manufacturing of formulated mRNA vaccines. In line with this, the Aspen Batch Process Developer V9.0 (Aspen Technology, Burlington, MA, USA) software was recruited to simulate the interdependent parameters of the biopharmaceutical processes’ conditions, converging the material system mass balances, while estimating cycle-time schedule and unit block size. Leveraging on empirical bibliographic data and on a recipe-driven, flowsheet execution by the process developer simulator engine, a resource commitment analysis, was prepared for the investigated continuous production process. Moreover, the parametrized data contributed towards the expenditure allocation assessment, committed for capital investment and operational requirements, delineated the manufacturing design of approximately 8000 g sterile, pyrogenic-free, formulated mRNA vaccine modalities of pharmaceutical grade quality. 

## 2. Materials and Methods

### 2.1. Comprehensive Overview of the Sheet Workflow and Methods Design Strategy 

**Figure 1:**
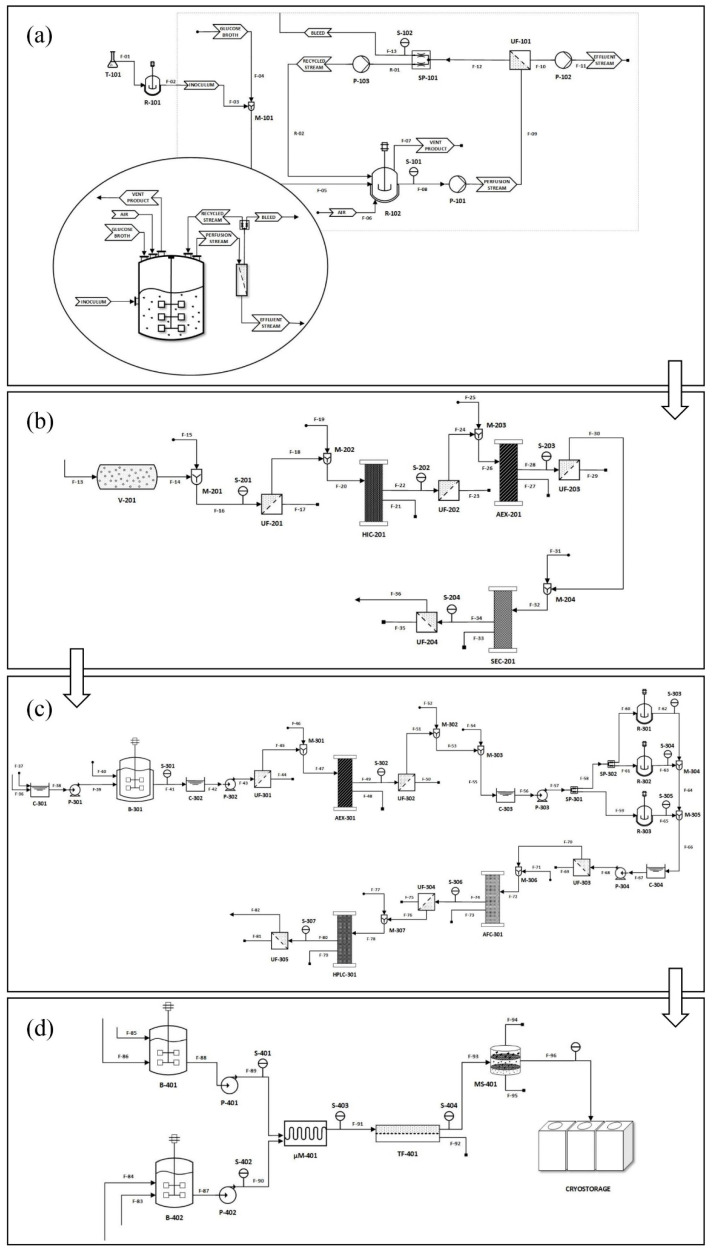
Process flow diagram of (**a**) perfusion bacterial culture upstream; (**b**) cell lysis and plasmid purification; (**c**) linearization and in vitro transcription; (**d**) lipid nanoparticle microfluidic formulation.

After inoculation transfer from flask T-101 to bioreactor R-101, the gene-transduced bacteria colony is streamed to the perfusion reactor system, compounded by the R-102 stirred bioreactor chamber, P-101, P-102, and P-103 pumps, UF-101 ultrafiltration device and SP-101 splitter, as displayed by Figure 1a. After cell lysis completion inside the V-201 vessel and filtration by UF-201, the plasmid DNA, embodying the genes of interest and the structural corresponding mRNA elements, i.e., the 3’ poly (A) tail) and RNA polymerase promoter, is obtained. Next, simulated moving bed (SMB) counter current, sorptive liquid chromatographic separation techniques of hydrophobic interaction HIC-201, ion-exchange AEX-201, and size exclusion SEC-201 are streamlined for the purification of the plasmid DNA population [10]. For the SMB inlet and outlet flows, moving in respect to stationary solid beds [11], buffer exchange is assisted by the various ultrafiltration devices UF-202, UF-203, and UF-204 (see Figure 1b). Linearization, performed by the sequence-specific restriction enzymes, is performed inside the B-301 reactor, hence the continuous operation is supported by the P-301 and P-302 pumps and C-301, C-302 accumulation and mass balancing vessels (see Figure 1c). The linearized template DNA is purified by AEX-301. The IVT step is sequentially performed as continuous in-buffer reaction, consuming recombinant RNA polymerase, nucleotide blocks and eventually the Cap analogue, by reactors R-301, R-302, and R-303; accumulation vessels C-303, C-304; and stream splitters SP-301, SP-302 (see Figure 1c). The SMB downstream protocols of affinity AFC-301 and high pressure HPLC-301 chromatography were employed to purify the mRNA product, emancipating the subsequent removal of cellular debris and DNA residues. The optimized material composition guided the vaccine self-assembly through the microfluidic μM-401 formulation, rendering the encapsulation of mRNA B-402 to the mixed lipid nanoparticle (LNP) carrier B-401, escorted by the tangential flow purification step TF-401 followed by the molecular sieve MS-401, before cryopreservation warehousing (see Figure 1d). Various quality control analytical stations (S-101 to S-405) were installed into the virtual process flow to monitor in real time the production conditions, progress, and the material attributes. A deterministic, multivariate factorial sensitivity analysis was implemented for the identified critical process parameters, to explore the design space of operations. The results obtained were subjected to Monte-Carlo uncertainty assessment, utilizing machine learning augmented analytics, in order to approach a realistic description of probable cost fluctuations, through projected lifecycles.

### 2.2. Inoculation, Upstream Production, and Plasmid Augmentation

We chose the mRNA sequence to encode for novel multiepitope, adjuvant-free peptidic constructs, eliciting cellular and humoral immune responses [12]. A sample of 10 mL of transformed *E. coli* colony T-101 was inoculated to 37 °C, inside fermenter R-101 of 1 L volume, containing lysogeny broth culture medium and 100 g/mL ampicillin antibiotic for 4–8 h, under mild stirring conditions. During fermentation, critical parameters (pH, O_2_ concentration, foaming, temperature) are monitored and the cell density is periodically measured by photometric quantification at 600 nm. Cell colonies are collected and centrifuged and the received cellular precipitate is stored at ≤−20 °C until medium insertion to the perfusion reactor R-102 (see detail inside the elliptic frame Figure 1a). Aeration provides oxygen enrichment while the distribution of nutrients is accomplished by low level mechanical stirring, i.e., to avoid wall rupture inhibited disturbance of cellular viability, linked to poor biomass density [13]. Cells are retained by the ultrafiltration system UF-101 and pumped through the separation device [14] SP-101, while the medium flows through as the reactor culture density increases [15]. The feed inlet flow of the fresh medium F-05 and the recycle stream R-01 to R-02 equilibrate the F-08 outlet to accomplish steady state, while the bleed F-13 run frequency is adjusted for the culture to reside for prolonged period inside the reactor vessel. The viable cell concentrations achieved require sparging to ensure adequate oxygen supply to F-06 flux in the system unit [14]. In order to design the main reactor vessel, the fluid inertia over gravitational force ratio, i.e., the dimensionless Froude number, was used, assuming uniformity of mixing conditions and O_2_ saturation, steady state operation, and identical production cycles:

(1) $$Fr=\;\frac{N^{2}D_{i}}{g}<0.1$$

where *g* is the acceleration of gravity (9.81 m/s^2^), *Di* is the internal vessel diameter in (m), and *N* is the rotational frequency in (Hz). The fluid dynamics in the vessel were described by the dimensionless Reynolds number for continuous impeller stirring:

(2) $$Re=\frac{\rho \;N\;D_{i}^{2}}{\mu }$$

where *ρ* is the fluid density in (kg/m^3^), *D_i_* is the internal diameter of the vessel in (m), *N* is the frequency rotation of impeller in (Hz), and *µ* is the fluid viscosity in (kg/ms). The Monod kinetic model was applied to calculate the biomass production, the limiting factor for glucose feed, nitrogen source was taken as the ammonium cation and the biomass yield to glucose mass input was assumed to be 0.4.

### 2.3. Alkaline Cell Lysis, Plasmid Purification, and DNA Linearization

**Figure 2:**
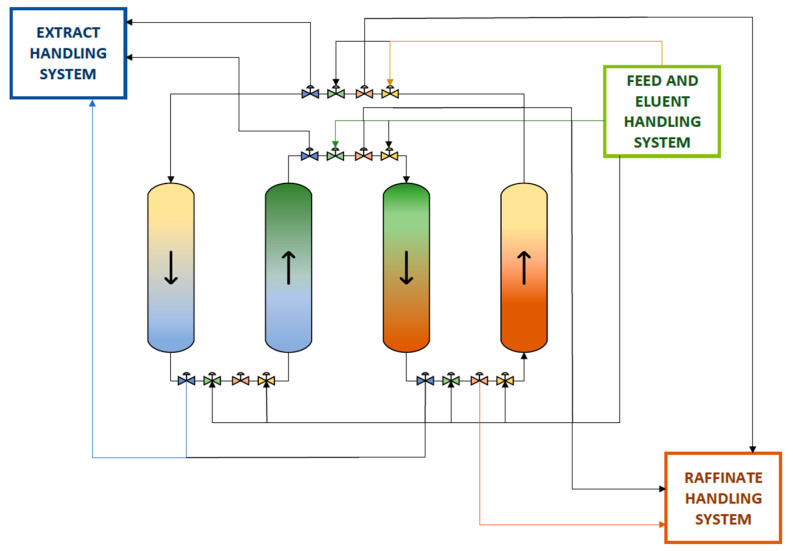
Schematic representation of periodic counter-current continuous chromatographic system applied principle of function. Colored streams represent inflows or outflows in open valves. After the passage of a ‘’switch time’’ period, valves open and close in series. Yellow stream indicates the mobile phase, blue stream the extract, green stream the feed solution, brown stream the raffinate.

Cells are subjected to alkaline solution for their wall structures to become ruptured and the desired polynucleotidic substance released [16]. Impact of excessive mechanical stress is cautioned to affect DNA distortion, constituting conventional stirrers, not suited. The proposed, scalable process is implemented as nondestructive and continuous [17]. SMB chromatographic devices provide high yields and purity of the desired final pharmaceutical product [11]. Chromatographic steps to assist the continuous operation were designed as four columns in series, packed with the desired resin, each of them connected with the inflow and outflow streams (feed, eluent, raffinate, and extract). Four valves were interposed between each column to determine the inflow/outflow type during switch time [10,18] (see Figure 2).

The switch time, being the retention time of the equivalent moving bed inside the column, was calculated by the Equation (3) [19]:

(3) $$t_{switch}=\frac{V\;\left(1-\varepsilon \right)}{\dot{V_{s}}},$$

where *V* is the volume of the packed part of the column in (m^3^), *ε* is the porosity, and $\dot{V_{s}}$ is the volumetric flow rate in (m^3^/min) of the equivalent true moving bed. The volumetric flow rates in the SMB conduct the same relevant movement in respect to the stationary resin as that in a true moving bed chromatography [10,11] (see Figure 2). For the chromatography types considered, 20 min retention time is assumed for a workable separation yield to be achieved [20,21,22]. Having obtained the plasmid DNA precipitate, HIC-201 was engaged in order to achieve lysate purification of RNA impurities or denatured plasmid forms, lipopolysaccharides (LPS) and contained protein debris [23]. The length of the stationary phase was approximated by the total length of the theoretical plates [24].

(4) $$H=A+\frac{B}{v}+Cv$$

where *H* is the length of one theoretical plate in (cm), *A* is the eddy diffusion parameter related to the mixing due to eddy motion, *B* is the longitudinal diffusion coefficient, relevant for gas systems and neglected in this case, *C* is the resistance to mass transfer coefficient between the mobile and the stationary phase, and *v* is the linear velocity of the mobile phase in (cm/h). The mathematical expression of the number of the theoretical plates *N* is:

(5) $$N=16{\left(\frac{t_{R}}{w}\right)}^{2}$$

where *t_R_* is the time of the peak formation and *w* is the peak width in (time units). The Van Deemter relation was used to correlate the height of the theoretical plate to the linear velocity of the mobile phase, based on bibliographic experimental values (see Table 1). 

AEX-201 conducts rapid, solvent free separation [25] of DNA due to the polyelectrolyte phosphate backbone binding to the cationic AEX resin moieties [26]. In addition, SEC-1 separates low molecular mass impurities like endotoxins and host cell proteins by pore-restraining them into the absorbent stationary phase, allowing plasmid DNA to depart from the column [16]. The absorbent in this case is a rigid allyl dextran/bisacrylamide matrix [21]. The Kozeny-Blake equation was utilized to determine the size of the column. The latter calculates the pressure drop inside the packed bed column of rigid particle elements under laminar flow [27]:

(6) $$\Delta P=\;\frac{\mu \;{Κ}_{0}{\left(1-\varepsilon \right)}^{2}L\;u}{d_{p}^{2}\;\varepsilon^{3}}$$

where, Δ*P* is the pressure drop inside the packed column in (psi), *μ* is the viscosity of the mobile phase in (cP), *K*_0_ is the empirical constant, *d_p_* is the particle diameter of the resin beads (in the case of porous membranes, the porous characteristic size) in (μm), *ε* is the porosity, *L* is the bed height in (cm), and *u* is the mobile phase linear velocity in (cm/s).

### 2.4. Design of IVT Reactor and Purification of mRNA

Synthetic mRNA fragments are prepared by IVT of the linearized template DNA, utilizing RNA bacteriophage polymerase (T7) in the abundant presence of structural ribonucleoside triphosphates NTPs (GTP, ATP, UTP, CTP) and RNase inhibitors. During the transcription reaction, each addition of a single nucleotide results in the formation of one pyrophosphate (PPi ≡ P_2_O_7_^4−^) ion, with the simultaneous release of one proton. The PPi species in turn bind to Mg^2+^ and form the precipitating magnesium pyrophosphate Mg_2_P_2_O_7_, a byproduct accumulation linked to pyrophosphorolysis, negatively affecting the RNA IVT production capacity [28]. The Michaelis-Menten equation was enforced utilizing the following reactions:

(RNA)_n_^v−^ + MgNTP^2−^ → (RNA)_n+1_^(v+1)**−**^ + MgP_2_O_7_^2−^ + H^+^

Mg^2+^ + MgP_2_O_7_^2−^ ⇌ Mg_2_P_2_O_7_ → Mg_2_P_2_O_7_ (solid)

IVT mRNA endures post transcriptionally capping by vaccinia virus enzymes, i.e., forming both diphosphate at the 5’ end by RNA triphosphatase and the subsequent generation of guanine (G) 5’ to 5’ triphosphate linkage, promoted by guanosyl transferase. The 5’ cap m7GpppG analogue structure is an essential element for the efficient translation of natural occurring mRNA. As criteria for the mixing effectiveness, we took into account the time of mixing *Θm*:

(7) Θm =5.9 (PV)−13     (dRDR)−13DR23

where *Θm* is the mixing time required to reach homogenous composition in (sec), *d_R_* is the impeller diameter in (m), *D_R_* is the vessel diameter in (m), *P* is the agitation power in (Watt), and *V* is the fluid vessel volume in (m^3^). In detail, the fraction of height to diameter (*H/D*) of the vessel was set to unity, height (*H*) of 230 mm, single agitator per axle diameter (*Di)* 120 mm, while the stirring was performed by a Rushton impeller at speed (*n*) of 200 rpm, mixing power (*P*) 80 W, mixing capacity (*P/V*) 10 W/L, for mixing time (*Θm*) of 0.48 s. For the processed material system Reynolds number (*Re*) 103,000, Froude number (*Fr*) 0.26, mass mixture (*M*) 4.2 kg and density (*ρ*) 517 kg/m^3^, were considered (see Equations (1) and (2)).

The IVT reaction is linked to the formation of double stranded (dsRNA) byproducts, which promote immunostimulatory effects in vivo [28]. The predominantly spurious product formed is a 3’-extended species of the run-off transcript, able to generate RNase III-sensitive dsRNA moieties [29]. The re-binding of RNA polymerase results in the back folding of the RNA, followed by self-extension. IVT RNA produced at high temperatures greater than 48 °C demonstrates a decrease of the 3’-extended byproducts biosynthesis. In addition, the poly-A tailing in high temperature reduces the formation of dsRNA byproducts, improving the RNA quality, alleviating the need for post-synthesis chemical purification.

The natural eukaryotic mRNA contains a 3’ polyadenylate tail of approximately 150 adenine bases that protects the molecule against restriction enzymes such as phosphatases and nucleases [30]. Thymine serial bases are therefore integrated to the plasmid DNA to construct the poly-adenine tails by the IVT step. These poly-A tails are then exploited by the affinity chromatographic step, as they form the substrate for binding to a poly (U)-Sepharose/oligo(dT)-cellulose resin [31]. The latter was considered in this study, hence the poly (U)-Sepharose resin requires intensified elution conditions, i.e., 1 g of oligo (dT)-cellulose binds up to 60 A_260_ (μg/mL) poly-A tail units [32]. Finally, the HPLC-301 purifies the IVT mRNA from dsRNA impurities [20]. 

### 2.5. mRNA Lipid Nanoparticle (LNP) Formulation Design

The RNA encapsulation to the lipid nanoparticle carrier was simulated using a staggered Y-shape herringbone micromixer [33]. Microfluidics offer a rapid, reproducible method of RNA formulation involving the lipid mixing of the organic solvent and lipid composition B-401 with the aqueous colloidal suspension containing the RNA constructs inside the B-402 vessel. The nucleic acid of negative charge interacts with the cationic lipidic groups fostering functional encapsulation efficiency [3]. LNPs of defined particle size distribution are produced by controlling the microfluidic flow rate (set up in this case to 200 mL/min). In order to estimate the surface coverage by the lipid moieties, for all *i* = 1, …, n mol, *j* = 1, …, n atoms (*i*) we let $a^{ij}=\left(a_{1}^{ij},a_{2}^{ij},a_{3}^{ij}\right)$ for the coordinates of the *j*-th atom of the *i*-th molecule. When the *i*-th molecule is rotated to the axes $x_{1},x_{2}$,$x_{3}$ the angles which define the said rotations $\theta^{i}=\left(\theta_{1}^{i},\theta_{2}^{i},\theta_{3}^{i}\right)$ are produced. For each molecule displacement the barycenter becomes $c^{i}=\left(c_{1}^{i},c_{2}^{i},c_{3}^{i}\right)$ thus transforming the coordinates $a^{ij}$ to $p^{ij}=\left(p_{1}^{ij},p_{2}^{ij},p_{3}^{ij}\right)$. Observing that $p^{ij}\;=\;c^{i}\;+\;R(\theta^{i})a^{ij}$, whereas $R\left(\theta^{i}\right)$ is the given rotation matrix, we wished for all atoms $\|p^{ij}-p^{i^{\prime }j^{\prime }}\|\geq d_{tol}\;\left(i\right)$, whereas *d_tol_* = 2 Å tolerance was set for constraints $g_{l}^{ij}\left(p^{ij}\right)\leq 0,l=1,\ldots ,r^{ij}\left(ii\right)$, which are in turn integrated into the objective function.

(8) $$\begin{array}{cc} f\left(c,\theta \right) & =\overset{nmol}{\underset{i=1}{\sum }}\overset{natom}{\underset{j=1}{\sum }}\left(\overset{nmol}{\underset{i^{\prime }=i+1}{\sum }}\overset{natom\left(i^{\prime }\right)}{\underset{j^{\prime }=1}{\sum }}max{\{0,d_{tol}^{2}-\|p^{ij}-p^{i^{\prime }j^{\prime }}\|\}}^{2}\right)\\ & +\overset{nmol}{\underset{i=1}{\sum }}\overset{natom\left(i\right)}{\underset{j=1}{\sum }}\left(\overset{r^{ij}}{\underset{l=1}{\sum }}max\;{\left\{0,g_{l}^{ij}\left(p^{ij}\right)\right\}}^{2}\right)\;, \end{array}$$

where nmol is the total molecule number of 1,2-Dioleoyl-sn-glycero-3-phosphoethanolamine (DOPE), dimethyldioctadecylammonium (DDAB), cholesterol (CHOL), 1,2-distearoyl-sn-glycero-3-phosphocholine (DSPC), polyethylene glycol (PEG), natom (*i*) the atom number of the *i*-th molecule $c=\left(c^{1}\;,\ldots ,\;c^{nmol}\right)\in IR^{3xnmol}$ and $\theta =\left(\theta^{1}\;,\ldots ,\;\theta^{nmol}\right)\in IR^{3xnmol}$. The function f(c,θ) vanishes whether the objectives (i) and (ii) are fulfilled, i.e., angles and displacements fit the region where *f* = 0 and are separated thus pertaining to the unconstrained minimization of f(c,θ) [34]. Molecules were represented numerically by the atomic cartesian coordinates assuming the origin as the barycenter, utilizing the PACKMOL solver, Institute of Chemistry and Institute of Mathematics University of Campinas, Brazil. In order to coat the RNA core with lipid monolayer, an approximation was made of the average area per lipid (*APL*) [35] by

(9) $$APL_{DOPE,DDAB,CHOL.,DSPC,PEG\;}=\frac{2\times X^{2}}{Lipid\;Number}\approx 0.65\;{\mathrm{nm}}^{2}$$

The VMD Molecular Graphics Viewer Software, University of Illinois, USA was used for visualization of the material system unit [36]. 

### 2.6. Clean Room Design

For the sterile mRNA production and formulation, the Directive 2003/94/EC for medicinal products of human use must be met and therefore manufacturing is retained within cleanroom facilities. The latter mechanically maintain defined sterile environments, imposing significant construction-related and operating costs. Critical elements of cleanroom design impacting costs have been developed as statistical model framework, rationalizing the expenditure evaluation demonstrated by the empirical equation [37]:

*LCC* = −1,645,051 + 5156.6*CA* + 68.8*MAV* + 34*EAV* + 514,211*TAV1* − 742,442*TAR* + 2,996,627*AFC*,

where *CA* is the cleanroom area in (m^2^), *AFC* is the average filter coverage rate (%), CL is the cooling load in (KW), *AAV* is the average airflow velocity in (m/s), *MAV* is the make-up air volume in (m^3^/s), *RAV* is the recirculated air volume in (m^3^/s), *EAV* is the exhaust air volume in (m^3^/s), *TAV* is the type of air ventilation, *TAR* is the type of air return, *THF* is the type of high efficiency filters, and *TC* is the type of chiller.

### 2.7. Resource Analysis Method, Total Capital Investment, and Production Cost

The total capital investment (CAPEX), i.e., the equipment and auxiliary’s procurement and installation cost (fixed capital investment), the direct working capital, and the operating cost (OPEX) consisting of personnel, raw material, utilities, and electricity expenditures were estimated. The cost of each device of the production unit was calculated:

(10) $$C_{p}\;=\;C_{p,0}{\left(\frac{X}{X_{0}}\right)}^{n}$$

where *C_p_* is the purchase cost of the device with characteristic size *X*, *C_p_*_,0_ is a previous known purchase cost of a similar device with a characteristic size *X*_0_, and *n* is the exponent associated with the device type, namely the Lang factor. Reference costs and sizes were retrieved from similar studies [38,39], while the Chemical Engineering Plant Cost Index (*CEPCI*) was utilized to adjust the equipment prices to inflation/deflation trends,

(11) $$C_{p,2}=C_{p,1}\;\frac{CEPCI_{2}}{CEPCI_{1}}$$

where *C_p_*_,1_ and *CEPCI*_1_ is the equipment price and the *CEPCI* value the initial year of operations, and *C_p_*_,2_ and *CEPCI*_2_ is the equipment price and the *CEPCI* value for the second year. It is assumed that each filtration membrane in every ultrafiltration and tangential flow filtration device is replaced for every perfusion cycle [39]. The total capital investment is calculated using the percentage of delivered-equipment cost for a solid-fluid processing plant (see Table 2) [40].

The cost of manufacture (€/year) considering no depreciation was calculated by the equation [41]:

*COM_d_* = 0.18*FCI* + 2.73*C_L_* + 1.23 (*C_U_* + *C_W_* + *C_R_*) (12)

where *FCI* is the fixed capital investment in (€), *C_L_* is the cost of labor in (€/year), *C_W_* is the cost of waste treatment which is considered negligible, and *C_R_* is the raw material cost in (€/year).

#### 2.7.1. Minimum Selling Price of Dose (MSPD) Estimation

A discounted cash flow analysis was performed in order to estimate the minimum selling price of the dose (€/dose), i.e., the selling price that generates the zeroed net present value (NPV):

(13) $$NPV\;(€)\;=\;-C_{0}\;+\;\sum_{0}^{n}\frac{C_{n}}{{\left(1+r\right)}^{n}}$$

where *C*_0_ is the total capital investment, *n* is the plant lifetime, *C_n_* is the cash flows in (€) and the year *n*, and *r* is the discount rate. It was assumed that the manufacturing unit is equity financed with no debt spanning the 30-year lifetime, out of which the first 3 years are devoted for the construction and start-up. The discount rate is assumed equal to 10% [41] and the active pharmaceutical ingredient RNA dose is assumed to approximate 30 μg, mirroring actual commercialized values. The annual cash flow for the year *n* was calculated using the equation:

*C_n_* = (*R_n_* − *COM_d,n_* − *d_n_*) (1 − *t*) + *d_n_* (14)

where *R_n_* is the annual revenues in (€), *COM_d,n_* is the manufacturing cost the year *n*, *d_n_* is the depreciation, and t is the income tax rate. The latter was set equal to 21% while a class life of 9.5 years was assumed [41] using a 5-year Modified Accelerated Cost Recovery System (MACRS) method of depreciation.

#### 2.7.2. Uncertainty Analysis

A Monte-Carlo uncertainty analysis was conducted utilizing the Oracle Crystal Ball, Austin, TX, USA software assuming a triangular distribution against maximum ±40% deviation of conversion. Elaborating on the former, the necessary information was generated running sensitivity iterations of the MSPD, thus projecting the parametric change probable fluctuation over time (see Table 3).

## 3. Results

### 3.1. Upstream Perfusion Reactor Design

The reactor’s working volume was set to 1000 L to compensate for the desired production flow, featuring 250 rpm mixing achieved by Rushton radial flow impeller [42]. The O_2_ supply was set at 30% medium saturation, pH was sustained in neutral values through the use of ammonia buffer 25% (*v*/*v*), and the temperature at 37 °C by external thermal jacket. Τhe culture was inserted in concentration of 5% (*v*/*v*) achieving maximum C_max_ value after 12 h, signaling the fermentation apex. Glucose carbon source in concentration of 8 g/L was infused and cellular density > 10^7^ cell/mL was documented by the S-101 supernatant optical density sensor [43]. Suspension pumping introduces complexity referring to the cellular viability condition [44], while filtering offers dual function removing metabolic debris between filtrate and medium, increasing the obtained culture density. Regarding supporting auxiliaries, peristaltic pumps P-101, P-102, P-103 were interposed between the vessel and the filter, after the filter and the splitter and the vessel, respectively, to regulate the assisted transfer of the perfusion liquid biomass, the recycling and effluent stream flux, respectively. The retentate stream splits into the product bleed and the recycle stream returns to the culture vessel (see Figure 1a). The sub-processes arrangement was designed in lieu of external separation options, hence recycling operation ensures accessibility for filter cleaning, unclogging, and control. Cellular death is found as a function of difference between pressure transmembrane difference, pore size, shear rate, and membrane cell tension [45]. High shear rates increase the transmembrane pressure difference at the filter inlet. Cells become damaged at shear rates exceeding 6000 s^−1^ and lose viability at a shear rate of 1300 s^−1^ [46], while for the related transmembrane pressure difference, cell damage occurs where cells occlude pores [15]. 

**Figure 3:**
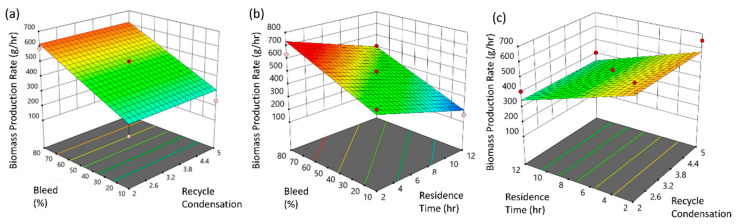
Surface response plots of biomass production rate in relation to: (**a**) bleed and recycle condensation; (**b**) bleed and residence time; (**c**) residence time and recycle condensation.

Elaborating the effect of pore size through recycling condensation, the culture viability state through production rate, bleed fraction, and residence time was investigated towards optimization (see Figure 3a–c). The ANOVA study, see Table 4, revealed the critical factors bearing *p*-values < 0.05, hence the proposed model appears significant, being assigned limited probabilities of 0.03%, that the displayed results could be attributed to noise. The predicted R^2^ value 0.73% is in agreement to the adjusted R^2^ of 0.83%.

The biomass production rate is found to increase when bleed is augmented while recycle condensation becomes slightly depressed, depicting the hazard of quality deterioration. Prolonged residence time high shear surface, owing to the increase of cell and debris accumulation [47], negatively affects the cell tolerance (see Figure 3b). Increasing residence time, shear stress induced culture death is increased, a phenomenon accompanied by lower mean cell diameter and significant residuals, which in turn emancipates the decreased biomass production rate [44] (see Figure 3b). Although the perfusion flux was calculated at 5 L m^−2^ h^−1^, i.e., an order of magnitude lower when compared to typical cross-flow filtration processes [48], filter fouling still remains a hurdle. Counterintuitively, pore membranes of 2 μm diameter require change every 5 days when operations are carried at a filter flux of 4–5 L m^−2^ h^−1^ [49], while replacement after 5 days is also advised for 5 μm membranes when the filter flux of 1 L m^−2^ h^−1^ [47].

Conclusively, no maintenance advantage of larger pore selection is claimed, especially when considering the poor cell retention capability, and therefore utilizing membranes carrying pores of 0.65 μm were organized as longitudinal clusters of 200 μm. This restricted pore radius results in the accumulation of nonviable cells and cell debris, pertaining to the decline of biomass production during prolonged bleed operations, leading also to a recycle condensation slight decrease (see Figure 3c).

### 3.2. Plasmid Deliverance by Alkaline lysis, Purification, and Linearization

**Figure 4:**
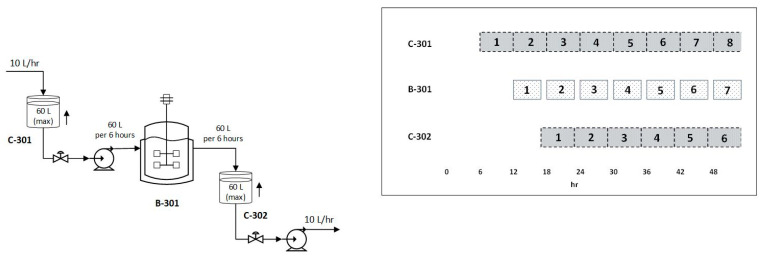
Flow rate and correlated operational time schedule for the alkaline cell lysis reactor B-301 and the auxiliary accumulation balancing vessels C-301, C-302.

Cell lysis takes place in a flow-through, stainless-steel tube container filled with glass beads protecting DNA from excessive shear force. The process is continuous, and the mixing time is set to 3 min to avoid denaturation. The flow rate and the volume of the tubular container is adjusted, as exhibited by Figure 4. The working volume of the stabilization vessels is designed to hold the material quantity until the upcoming cycle is completed, maintaining the inlet and outlet flow. For the 10 L/h material volume flow to be processed in a 6-h cycle reactor schedule, the stabilization vessel accumulates the material inserted during the completion of each cycle, showcasing the volumetric adaptation design of 60 L, as demonstrated by Figure 4.

A neutralization, automated mixing step of the ammonium sulfate with the lysed cell solution using a connector (Y or T) was considered. During mixing, a precipitate that consists of genomic DNA, cell debris, and proteins is formed. The clarified lysate becomes precipitate-free via a passage through the ultrafiltration device UF-201. HIC-201 separates *E. coli* lysate from impurities such as RNA and denatured plasmid constructs. The number of the theoretical plates, indicative of the separation, was estimated for the scale up transfer [24]. Linear velocity of the stationary phase ascended as the critical scaling parameter. Parameters *A* and *C* being system-specific were estimated by experimental bibliographic data for packed hydrophobic interaction chromatography of different sizes [23]. The calculation used the least square method for the linear velocity and plate heigh correlation, *A* = 2.75 and *C* = −0.73, respectively. Fitting of the values to experimental data was conducted and the linear velocity of the mobile phase was determined by the Kozeny-Blake equation, assuming negligible pressure drop inside the packed column. The bead particle size was found, approximately *d_p_* = 30 μm, the porosity *ε* = 0.3, and the viscosity of the mobile phase (ammonium sulfate aqueous solution) was assumed as equal to that of the water *η* = 1 cP [23]. For the perfusion culture size capacity of 40 tons *E. coli*/year, HIC-201 columns of dimensions 38 cm length and 19 cm diameter, typical in the pharmaceutical technology, were resolved, all featuring the said retention time of 20 min.

AEX-201 was utilized to separate the plasmid DNA from the *E. coli* lysate suspension. The colloidal dispersion obtained by HIC-201 enters the iterated anion exchange columns containing thick packed membranes of porous-bearing average diameter size 0.8 μm. The Kozeny-Blake empirical constant, given the mobile and stationary phase types, was assumed equal to 150 [50]. For mobile phase’s viscosity set to 1 cP, each column features 43 cm length and 21 cm diameter, for the given 20 min retention time. SEC-201 was used for ongoing purification of the plasmid DNA. Table 5 shows the iterated parameters of the Kozeny-Blake equation.

### 3.3. Purification of the Linearized DNA Template and IVT

**Figure 5:**
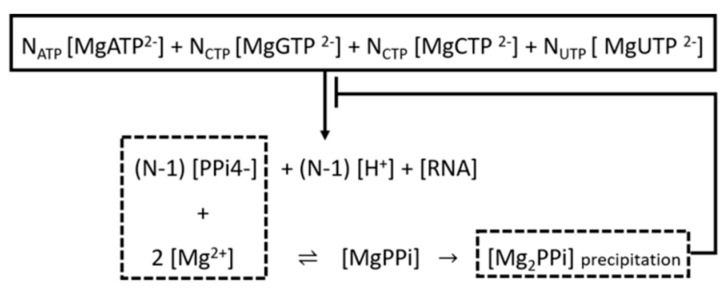
Schematic diagram of the IVT kinetic model, applied to the Aspen V9.0 simulator engine.

The linearized DNA template is captured from the concentrated solution via AEX-301 [51], entering the four chromatographic columns of 7 cm length, 3.5 cm diameter, featuring 5 min retention for each. Elaborating on the applied kinetics of the IVT reaction the NTP^4−^ ions bind to Mg^2+^ to form the variety of complexes Mg_2_NTP, MgNTP^2−^, MgHNTP^−^, whilst RNA polymerase requires ions Mg^2+^ and MgNTP^2−^ in order to progress, as demonstrated by Figure 5. 

Consequently, the Michaelis-Menten pattern was enforced given by the relation:

(15) $$V=k_{app\;}\left[T7RNAP\right]\;\frac{\left[MgNTP^{2-}\right]\times \left[Mg^{2+}\right]}{\left[MgNTP^{2-}\right]+k_{1\;}\left[Mg^{2+}\right]+k_{2}}\;,$$

where [*MgNTP*^2−^] is the lowest of values amongst concentrations of [*MgATP*^2−^], [*MgCTP*^2−^], [*MgGTP*^2−^], and [*MgUTP*^2−^], and the obtained by experimental and prediction studies parameters are *K_app_* = 2.04 min^−1^, *K*_1_ = 0.23 mM, *K*_2_ = 0.094 mM. The resulting 5’ end guanine methylation is induced either on the first (m7GpppNmpN …; cap1) nucleotide or on both the first and the second (m7GpppNmpNm …; cap2) nucleotide following 5’ cap thus enhancing the translation efficiency.

For our IVT reactor design, the transcription protocol was performed at 48 °C, contemplating documented functional volume restrictions of 20 L [52]. Taking into account the reaction’s scalability limiting factor, we choose to incorporate three 10 L vessel reactor units. The RNA production rate response was statistically coded as quadratic and the relevant ANOVA [53] of results are demonstrated by Table 6. The projected F-value of 23.96 appoints significance to the model, hence only a 1.21% chance exists that the latter could be appointed to noise.

**Figure 6:**
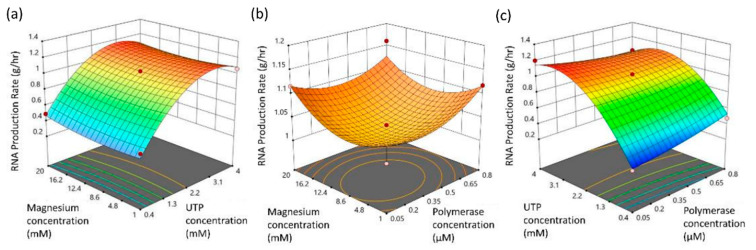
Surface response plots of biomass RNA IVT production rate against factorial concentration projections: (**a**) magnesium and UTP; (**b**) magnesium and polymerase; (**c**) UTP and polymerase.

The sensitivity analysis study showed that maximization of RNA production rate occurs when UTP concentration is valued between (2.2–4) mM, as demonstrated by Figure 6a, and in the semi ovoid operational space as projected by Figure 6b, defined by the Magnesium concentration range of (4.8–12.6) mM and finally for polymerase concentration values threshold (0.3–0.5) μM. Since magnesium is a sub-process derived byproduct, the optimal design operational space is defined by the UTP and polymerase concentration depicted for the final graph presentation of Figure 6c. The calculated p-values (see Table 6) indicate that UTP-concentration and its squared term (1.2, 7.9) 10^−3^, respectively, ascend as the key process-related, material parameters. 

**Figure 7:**
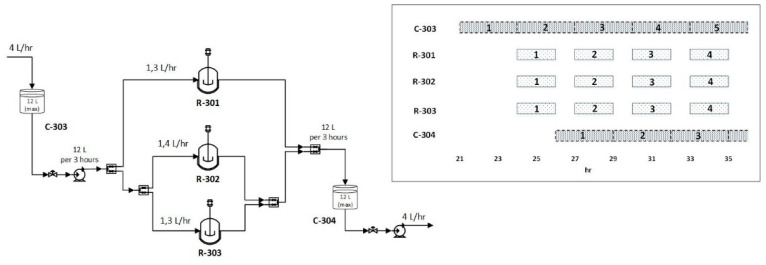
Flow rate and correlated operational time schedule for the IVT reactors R-301, R-302, R-303 and their auxiliary, accumulation vessels C-301, C-302.

The schedule of operation for the IVT reactors R-301, R-302, and R-303 and the supporting mass equilibration vessels C-303 and C-304 is projected by Figure 7. Assuming 80% of occupancy filling, a repeatable 4-h cycle process time with 80 min chronic intervals between subsequent launches is considered.

Poly-A tails form the basis for binding in a poly (U)-Sepharose or a oligo (dT) -cellulose resin [31]. The latter was chosen for this study, hence the poly (U)-Sepharose resin requires stronger elution conditions. According to bibliography, 1 g of oligo (dT)-cellulose is capable of binding up to 60 A_260_ (μg/mL) units of poly-A tails [32]. The final chromatographic step HPLC-301 purifies the in vitro transcribed mRNA from dsRNA impurities [20]. Pertaining to this scalable, fast and efficient process, an alkylated non-porous matrix of polystyrene-divinylbenzene copolymer microspheres with a diameter size 2.1 μm is used. Purification of mRNA is performed by chromatographic steps AEX-301, AFC-301, and HPLC-301 and their related designed specifications are given by Table 7.

### 3.4. LNP Formulation and Self-Assembly

**Figure 8:**
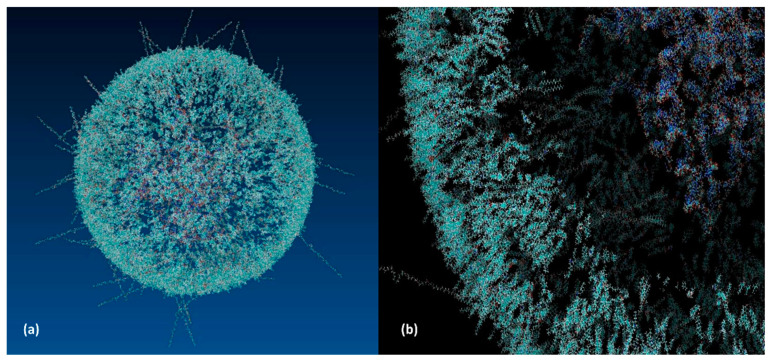
Molecular graphics of the: (**a**) lipid nanoparticle unit; (**b**) the made-transparent surface section, showing the encapsulated mRNA constructs occupying the nuclear area.

Regarding the lipid shell assembly, 620 DSPC, 2876 Cholesterol, 2280 DDAB, and 124 PEG molecules were estimated to fulfill the minimum requirement for encapsulation of 22 RNA antigenic constructs, inside a nanosphere of 250 Å in diameter. These results are in line with experimental work [33] assessing the scalability and robustness of microfluidic operating key parameters on the lipid nanoparticle production. The latter, in combination with the desired microfluidic flux of 200 L/min [33], fed the process parameters of the mass balance flow-sheeting, facilitating the evaluation of raw materials consumption. In Figure 8a,b, the lipid sphere unit and a transparent section of the nanoparticle are displayed respectively using the VMD molecular visualization console (VMD-Visual Molecular Dynamics software, Theoretical and Computational Biophysics Group, University of Illinois).

### 3.5. Clean Room Related Costs

For gene therapy product manufacturing, Grade A/B of cleanliness must be maintained utilizing negative gradient pressure surroundings of containment conditions. The proposed regression model calculated the cleanroom life cycle cost. The floor area change *CA*, *EAV*, *MAV*, and cleanliness level, reflected by the *AFC*, affect the cost positively. According to the *TAV1* coefficient, *FFU* ventilation was found to cost less than plenum or filter, while the negative *TAR* demonstrates that raised floor air return overrides the cost of wall vents. Conclusively, the clean room life cycle expenditures were included to the utilities cost (C_u_).

### 3.6. Resource Commitment Analysis

The referenced costs [38,39] and the equipment sizing required for producing 8000 gr of API RNA corresponding to roughly 270 million doses are shown by Table 8. 

Towards the overall production expenditure estimation, the utilities (C_U_), labor (C_L_), and the raw material (C_R_) costs were considered. The raw material necessary for the desired annual production and their corresponding prices are listed by Table 9. The utilities cost was associated with the mechanical operation of the perfusion, the IVT transcription reactor units, and the media transfer through the pumps. A consensus of a 31-day turnaround with 30 days perfusion functionality and 1 day reserved for cleaning and sterilization was reached, corresponding to 11 completed cycles per annum. Labor cost was calculated using the number of operators [56] and assumed approximately 190,476 € per operator [57].

### 3.7. Uncertainty Analysis

**Figure 9:**
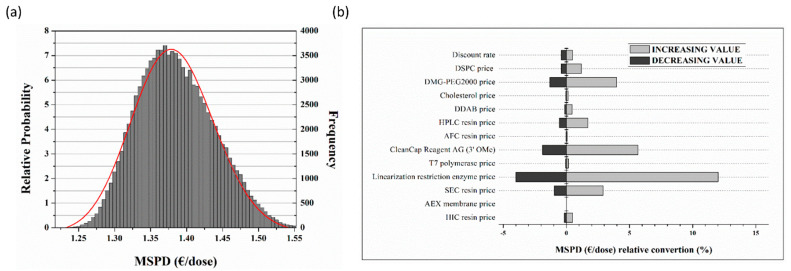
MSPD Monte-Carlo related data (**a**) Complex factorial, relative probability density function of MSPD against relative probability and Monte-Carlo frequency occurrence; (**b**) relative conversion against baseline scenario for the participating process wide raw materials.

The uncertainty analysis displayed by Figure 9a generated the sensitivity plot of the Minimum Selling Price of Dose (MSPD) (€/dose), emancipating the parametric changes likely to fluctuate over time. On the y_1_ axis a relative probability number assigns a real, nonnegative number of y_1_ to any ordered sum of events, while the y_2_ axis denotes the frequency of the said events occurring, post 10^5^ iterated random walks. Fitting the distribution, the following equation is obtained marked by Figure 9a by the indicated characteristic red Gaussian curve.

(16) $$y=-0.19+\frac{1.069}{\left(0.041\sqrt{2\pi }\right)x}e^{\frac{-\ln{\left(\frac{x}{1.38}\right)}^{2}}{0.0033}}$$

Conclusively, the MSPD price correlated to single dose regime ranges from 1.25 to 1.55 Euros featuring a rather reserved dispersion and therefore limited dependence borne by the various fluctuations considered. Expanding on this, several indicators namely tax rate, discount rate, lipid carrier ingredients DSPC, PEG, cholesterol, DDAB, resins for HPLC, AFC, SEC, HIC, and enzymes T7 and linearization restriction and their possible deviations assumed from the baseline are displayed by Figure 9b.

## 4. Discussion

A deterministic, multivariate, factorial sensitivity analysis was implemented, for the identified critical process parameters, exploring the operating and the material quality attributes design space, through model equations. Regarding upstream a perfusion flux of 5 L m^2^ h^−1^, pore membranes of 2 μm diameter, and volume of main reactor chamber of 1000 L operating with 250 rpm, were resolved as appropriate solution. Moreover, for the IVT reactor system, three 10 L units (8 L of actual working volume for each) with respective input concentrations of 4 mM UTP, 10 mM Mg^2+^, and 0.5 μM polymerase were investigated, respectively. Regarding the purification steps conducted by chromatographic blocks, retention time of 20 min and the 10 L/h material flow was considered towards sizing of the equipment, 43 and 21 cm for length and diameter, respectively. Initial atomic configurations of spatial molecular arrangement of mRNA and lipid molecular species were orientated to keep the appropriate Van der Waals distance tolerance, thus elucidating the overall structure of the formulated substance composition. The actual number of molecules participating in the formation of a typical structural vaccine unit were estimated for the formulation step guiding the simulation. Clean room facilities featuring Grade A/B good manufacturing certification were considered to match the proposed solution.

**Figure 10:**
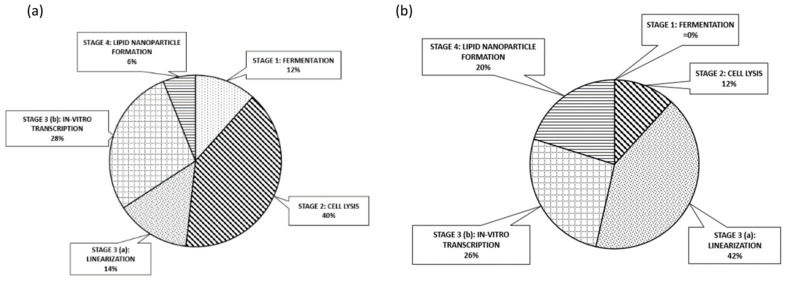
Percentile contributions to the overall cost (pies) of continuous GMP mRNA formulated products by lipoparticle carriers: (**a**) equipment; (**b**) raw material.

The results obtained, either by the empirical data and design equations or by the convergence of the mass and energy balances simulation, fueled our resource commitment analysis, regarding the procurement of equipment and raw material for GMP operations through the probabilistic Monte-Carlo assessment. The pricing fluctuations associated with product supply and demand through a projected 30-year lifecycle were concluded. The equipment resources allocation for each of the manufacturing steps is displayed by the pie chart of Figure 10a. Fermentation accounts for 12% of resource participation, cell lysis 40%, linearization reactors and related auxiliaries 14%, IVT reactors 28%, formulation associated pumps and microfluidic mixing devices 6%. Raw materials were also grouped according to their designated process step implementation and the results are herein presented by the pie of Figure 10b. Fermentation raw materials appear negligible, cell lysis reagents account for 12%, linearization reagents for 42%, IVT reagents for 26%, and LNP formulation-associated and primary carrier ingredient materials for 20%.

Kis et al. [52,63] investigated the technoeconomic feasibility assessment of the fed-batch campaign production of several available mRNA vaccine technologies starting from PCR constructs. Schmidt et al. [64] studied the combination of digital twin and PAT implementation for the batch production of lyophilized mRNA, while proposing the future implementation by continuously operating unit blocks that reduce the parallel work burden and ease the process control advancement. The present study struggled towards the investigation of such end-to-end formulated mRNA vaccines process transition from batch-to-continuous operations, performed in sustained clean room environment. 

In general, continuous production transition is linked to higher material utilization, minimization of waste, and improved energy consumption when compared to the similar process of batch operations, offering significant financial gains. These differences become increasingly apparent, as a rule of thumb, with the scale-up intensification of the studied manufacturing lines. For processes related to pharmaceutical products containing high potent APIs, as is the case of IVT mRNA formulated products, (whereas solely small-scale equipment is used, i.e., our IVT transcription main reactor unit is operating at only 8 L working volume), the gain appears nonexistent.

However, when quality assurance remains the higher of objectives, as herein, the intrinsic advantages offered by continuous manufacturing remain unparalleled [8]. Advocating the former statement, the dynamic nature of batch processes constitutes dependencies related to unavoidable human operator errors, hence numerous, chronically intensive, complex tasks must be performed on a recipe-call basis. Continuous bioprocessing on the other hand is carried out in steady state, under flow operations, allowing for automation adoption, material traceability, and real time analytical technology integration. In general, the proposed methodology rationalizes the operational roadmap as an orchestrated, modular, streamlined, controlled, and therefore quality by design-driven environment. This, systems-based approach, is therefore suited to support Smart Good Manufacturing Practices, enabling the 4.0 industrial principles’ introduction. Moreover, due to the iterated merits, technology transfer and decentralization becomes feasible, hence quality standardization is preserved, regardless of human intervention and lab lore. Under this lens, the desired critical material attributes can be tuned through an access control unit, constantly in connection with the sensing data, enabling both safety and repeatability. 

Several limitations have been contemplated for this study. For the perfusion reactor, glucose was considered the limiting factor in the Monod kinetic model and the O_2_ was assumed to homogeneously disperse into the working vessel volume. Moreover, the reaction producing biomass, water, and CO_2_ does not take into account byproducts. This fact is counterbalanced by our imported experimentally validated kinetic data and by the developmental curve fitting notations having embodied the yield corrections. Volume loss inside the chromatographic columns and the piping was deemed negligible, while the rest of the circulating impurities and residuals like bacteria cells and plasmid-DNA debris, respectively, were considered negligible and subsequently were not included in the transferred suspension mass. Moreover, aqueous solutions were correlated to the water’s viscosity. For the plasmid DNA-based IVT system the Mg^2+^ precipitation was not modelled separately but instead participated to the Michaelis-Menten equation. We furthermore assumed one linear DNA template provided by each experimental cell unit to partially counterbalance for the inevitable material reactor wall loss. 

## 5. Conclusions

Continuous, smart manufacturing 4.0 of biopharmaceuticals, and specifically of mRNA drug products, pertains to unmatched standardized quality assurance. Since lab scale experiments are resource-intensive, the application of in silico, plant-wide simulation tools empowers the field to evolve following a risk abatement strategy. For the envisioned batch-to-continuous operational shift, a simulation framework in order to design the process conditions is necessary. Our key findings from our study have been reached:

(a) We demonstrated the feasibility of continuous end-to-end GMP compliant, mRNA LNP formulated vaccine technology adoption, launching from bacterial culture. Innovations such as perfusion reactors, SMB chromatographic steps, continuous cell lysis modules, and microfluidic formulation were introduced to approach the task. This paves the way for the future actual, modular process line development implementation, under strict quality directives.
(b) The material-driven flowsheet converging mass and energy balances combined or leveraged by bibliographic, empirical, and experimental elements allowed us to link the operational design space framework to the critical quality attributes, hereafter offering a comprehensive mapping of the whole process, accessible for further experimental optimizations. Regarding the processes and the representative material system studied, bleed fraction, residence time, and UTP concentration were identified as the critical parameters for the component blocks of the perfusion bioreactor and the IVT reactors, respectively.
(c) This digital approach furthered allowed us to safely assess the economic aspects of the venture, documenting certain remarkable findings. The MSPD price correlated to single dose regime is expected to range most probably between 1.30 to 1.45 Euros. Indeed, the continuous production of mRNA vaccines overall cost conveys marginally the same price, when compared to batch, being attributed to the limited, applied scale-up grade. MSPD featured reserved dispersion and therefore limited dependence borne by fluctuations. Finally, cell lysis devices including their related purification modules and linearization enzymes ascend as the principal cost factors accounting for 40% and 42% of the equipment and raw material, respectively.

mRNA drug products have demonstrated safe administration and efficacy, thus offering a needed promising vaccination alternative due to their potency, adaptive pre-clinical to bedside trial design, and rapid and low-cost development and manufacturing [65]. Functional immunity by formulated mRNA has been elicited in vivo against several infectious diseases such as Zika virus, influenza virus, rabies virus, and others, by utilizing various sequence-optimized mRNA constructs encapsulated into LNP carriers [66]. Challenges still exist and include innovative measures to encumber the establishing of oriented regulation, documenting and increasing safety and efficacy, scaling-up, and decentralizing of sterile production in compliance with pharma industry 4.0 principles. This work delved into a plug-and-play model development investigating the annual, continuous GMP production of roughly 270 million doses (each containing 30 μg API mRNA). The latter methodology may be applied universally, i.e., independently of the targeted product use (proteins, monoclonal antibodies, antigens, derivatives), therefore contributing to the comprehensive process mastering, for any of the emerging RNA vaccine manufacturing platforms. 

## Figures and Tables

**Table 1:** Experimental data of (HIC-1) for A and C Van Deemter coefficients estimation.

**Experiment**	**Volume (mL)**	**Diameter (cm)**	**Plate Area (cm^2^)**	**Length (cm)**
1	28	1.6	2	14
2	334	5	20	17
**Experiment**	**Volume Feed (mL)**	**Retention Time (min)**	**Flow Rate (mL/min)**	**Linear Velocity (cm/h)**
1	0.5	40	0.0125	0.4
2	5	50	0.1	0.3
**Experiment**	**Plate Height (cm)**	**Peak Time (min)**	**Peak Width (min)**	**Number of Plates**
1	0.08	7	5	157
2	0.1	7	5	157

**Table 2:** Factors for total capital investment estimation.

Direct Costs	
Purchased equipment	100
Purchased equipment installation	39
Instrumentation and controls (installed)	26
Piping (installed)	31
Electrical systems (installed)	10
Buildings (including services)	29
Yard improvements	12
Service facilities (installed)	55
Total direct plant cost	302
Indirect Costs	
Engineering and supervision	32
Construction expenses	34
Legal expenses	4
Contractor’s fee	19
Contingency	37
Total indirect plant cost	126
Total Capital Investment	
Fixed-capital investment	428
Working capital (15% of total capital investment)	75
Total capital investment	503

**Table 3:** Uncertainty analysis parameters.

Parameter	Lowest Conversion	Lowest Value	Highest Conversion	Highest Value
HIC resin price (€/L)	−10%	1857	+30%	2683
AEX membrane price (€/m^2^)	−10%	890	+30%	1285
SEC resin price (€/L)	−10%	9802	+30%	14,159
Linearization restriction enzyme price (€/unit)	−10%	0.00459	+30%	0.00663
T7 polymerase price (€/mg)	−10%	34,200	+30%	49,400
CleanCap Reagent (3’ OMe) AG (€/mg)	−10%	223	+30%	322
AFC resin price (€/L)	−10%	9802	+30%	14,159
HPLC resin price (€/L)	−10%	9802	+30%	14,159
Dimethyldioctadecylammonium (DDAB) price (€/g)	−10%	498,600	+30%	720,200
Cholesterol price (€/g)	−10%	10	+30%	14
1,2-Dimyristoyl-*rac*-glycero-3-methoxypolyethylene glycol-2000 (DMG-PEG2000) price (€/g)	−10%	149	+30%	215
1,2-dioctadecanoyl-*sn*-glycero-3-phosphocholine (DSPC) price (€/g)	−10%	154	+30%	223
Discount rate (-)	−40%	0.06	+40%	0.4
Income tax rate (-)	−40%	0.126	+40%	0.294

**Table 4:** Design of experiment statistical models for the response biomass production rate (g/h) against factorials bleed (%) and residence time (h).

Source	Sum of Squares	df	Mean Square	F-Value	*p*-Value	Model A
Biomass Production	2.95 × 10^5^	3	98,287.81	19.4	0.0003	significant
A-Recycling Condensation	0.0005	1	0.0005	9.84 × 10^8^	0.9998	
B-Bleed	1.89 × 10^5^	1	1.89 × 10^5^	37.38	0.0002	
C-Residence time	1.06 × 10^5^	1	1.06 × 10^5^	20.81	0.0014	
Residual	45,601.9	9	5066.88			
Cor Total	3.41 × 10^5^	12				
Coded model equation: *Biomass Production Rate =* 464 − 0.0079 × *A +* 153.87 × *B* − 114.82 × *C* (14)						

**Table 5:** Design specifications for chromatographic columns.

Chromatography Block	ΔP (bar)	u (cm/s)	L (cm)	k_O_ (-)	η (cP)	ε (-)	d_p_ (μm)	N (-)	L_c_ (cm)	D (cm)
HIC-201	-	-	-	-	-	-	-	4	42.8	21.4
AEX-201	0.5	0.004	9	150	1	0.4	0.8	4	12.8	6.4
SEC-201	0.003	0.007	30	180	1	0.3	47	4	42.8	21.4

**Table 6:** ANOVA of RNA production rate exploring the design space of the factorials polymerase and UTP concentration.

Source	Sum of Squares	df	Mean Square	F-Value	*p*-Value	Model RP
Model RNA Production Rate	1.22	9	0.1355	23.96	0.0121	significant
A-Polymerase concentration	0.0017	1	0.0017	0.3032	0.6202	
B-UTP concentration	0.8282	1	0.8282	146.47	0.0012	
C-Magnesium concentration	0.0016	1	0.0016	0.2916	0.6267	
AB	0.0317	1	0.0317	5.6	0.0988	
AC	0.0004	1	0.0004	0.0716	0.8063	
BC	0.001	1	0.001	0.1843	0.6966	
A^2^	0.0022	1	0.0022	0.3829	0.5799	
B^2^	0.2271	1	0.2271	40.16	0.0079	
C^2^	0.0042	1	0.0042	0.7374	0.4536	
Residual	0.017	3	0.0057			
Cor Total	1.24	12				
Coded model equation: *RNA production rate =* 1.03 + 0.0146 *× A +* 0.3218 × *B +* 0.0144 × *C* − 0.08890 × *AB −* 0.01001 × *AC* − 0.0161 × *BC* + 0.0308 *× A*^2^ − 0.3152 *× B*^2^ + 0.0427 *× C*^2^						

**Table 7:** Kozeny-Blake equation parameters and chromatographies’ design specifications [20,26,27,51,53,54].

Chromatography Block	ΔP (bar)	u (cm/s)	L (cm)	k_O_ (-)	η (cP)	ε (-)	d_p_ (μm)	N (-)	L_c_ (cm)	D (cm)
AEX-301	0.1	0.002	5	150	1	0.4	0.8	4	7.2	3.6
AFC-301	-	-	-	-	-	-	-	4	23.4	11.7
HPLC-301	0.5	0.003	25	150	1	0.3	2.1	4	35.6	17.8

(ΔΡ) pressure drop inside the chromatography column, (u) linear fluid velocity, (L) resin height, (k_0_) Kozeny-Blake constant, (η) fluid viscosity, (ε) porosity, (d_p_) particle diameter, (N) number of columns, (L_c_) chromatography column height, (D) chromatography column diameter.

**Table 8:** Equipment sizing and cost.

Unit Block	Description	Size	*C_p_*_,0_ (€)	Year	Lang Factor	References	Simulation Size	*C_p_* (€)	*C_p_*_,2021_ (€)
R-101	Fermentation reactor	V = 0.065 m^3^	34,013 *	2013	0.6	[38] & assumptions	V = 0.1 m^3^	44,045	49,519
R-102	Perfusion reactor	V = 2 m^3^	594,299 *	2016	0.6	[39]	V = 1 m^3^	392,091	461,650
V-201	Horizontal vessel	V = 2.5 m^3^	12,450	2013	0.2	[38] & assumptions	V = 0.0005 m^3^	2267	2548
UF-201	Ultrafiltration filter	A = 1 m^2^	6686 *	2015	0.58	[39]	A = 0.023 m^2^	750	859
HIC-201	Chromatography	-	-	2016	-	[55]	-	500,000	588,702
UF-202	Ultrafiltration filter	A = 1 m^2^	6686 *	2015	0.58	[39]	A = 0.0016 m^2^	160	183
AEX-201	Chromatography	-	-	2016	-	[55]	-	500,000	588,702
UF-203	Ultrafiltration filter	A = 1 m^2^	6686 *	2015	0.58	[39]	A = 0.0016 m^2^	160	183
SEC-201	Chromatography	-	-	2016	-	[55]	-	500,000	588,702
UF-204	Ultrafiltration filter	A = 1 m^2^	6686 *	2015	0.58	[39]	A = 0.0016 m^2^	160	183
C-301	Container	V = 0.21 m^3^	271 *	2002	0.2	[40]	V = 0.075 m^3^	221	356
P-301	Pump	-	-	2021	-	Assumption	-	500	500
B-301	Jacketed mixer	V = 0.2 m^3^	29,931	2020	0.16	[55], Pall Corporation	V = 0.075 m^3^	25,584	27,369
C-302	Container	V = 0.21 m^3^	271 *	2002	0.2	[40]	V = 0.075 m^3^	221	356
P-302	Pump	-	-	2021	-	Assumption	-	500	500
UF-301	Ultrafiltration filter	A = 1 m^2^	6686 *	2015	0.58	[39]	A = 0.0028 m^2^	221	253
AEX-301	Chromatography	-	-	2016	-	[55]	-	500,000	588,702
UF-302	Ultrafiltration filter	A = 1 m^2^	6686 *	2015	0.58	[39]	A = 0.00047 m^2^	79	90
C-303	Container	V = 0.21 m^3^	271 *	2002	0.2	[40]	V = 0,05 m^3^	203	328
P-303	Pump	-	-	2021	-	Assumption	-	500	500
R-301	Reactor	V = 0.4 m^3^	74,699 *	2002	0.5	[40]	V = 0.01 m^3^	11,811	19,042
R-302	Reactor	V = 0.4 m^3^	74,699 *	2002	0.5	[40]	V = 0.01 m^3^	11,811	19,042
R-303	Reactor	V = 0.4 m^3^	74,699 *	2002	0.5	[40]	V = 0.01 m^3^	11,811	19,042
C-304	Container	V = 0.21 m^3^	271 *	2002	0.2	[40]	V = 0.05 m^3^	203	328
P-304	Pump	-	-	2021	-	Assumption	-	500	500
UF-303	Ultrafiltration filter	A = 1 m^2^	6686 *	2015	0.58	[39]	A = 0.006 m^2^	344	394
AFC-301	Chromatography	-	-	2016	-	[55]	-	500,000	588,702
UF-304	Ultrafiltration filter	A = 1 m^2^	6686 *	2015	0.58	[39]	A = 0.005 m^2^	309	354
HPLC-301	Chromatography	-	-	2016	-	[55]	-	500,000	588,702
UF-305	Ultrafiltration filter	A = 1 m^2^	6686 *	2015	0.58	[39]	A = 0.005 m^2^	309	354
B-401	Non-jacketed mixer	V = 0.2 m^3^	24,994	2020	0.16	[55], Pall Corporation	V = 0.0025 m^3^	12,398	13,263
B-402	Non-jacketed mixer	V = 0.2 m^3^	24,994	2020	0.16	[55], Pall Corporation	V = 0.0075 m^3^	14,780	15,812
P-401	Pump	-	-	2021	-	Assumption	-	500	500
P-402	Pump	-	-	2021	-	Assumption	-	500	500
μM-401	Microfluidic mixer	-	176,671 *	2021	-	Precision Nano-systems Corporation	-	176,671	176,671
TF-401	Tangential flow filtration filter	A = 0.12 m^2^	4504 *	2018	0.8	[39]	A = 3 m^2^	59,149	62,553
MS-401	Molecular sieve	-	-	2021	-	Assumption	-	1800	1800

* Price after unit conversion.

**Table 9:** Raw material quantification and pricing.

Raw Material	Price	References	Total Quantity	Total Price
Glucose	0.24 * €/kg	[58]	18,454 kg/year	4429 €/year
Diammonium phosphate	0.83 * €/kg	[59]	6336 kg/year	5259 €/year
Ammonium sulfate	0.124 * €/kg	[38]	21,384 kg/year	2652 €/year
HIC resin	2064 €/L (replacement every 100 cycles)	[55,60,61]	2206 L/year	4,553,070 €/year
AEX membrane	989 €/m^2^ (replacement every 100 cycles)	[55,60,61]	6 m^2^/year	5505 €/year
HPLC resin	10,892 €/L (replacement every 100 cycles)	[55,60,61]	1476 L/year	16,076,592 €/year
SEC resin	10,892 €/L (replacement every 150 cycles)	[60,61]	2546 L/year	27,736,042 €/year
Linearization restriction enzyme	0.0051 * €/unit	[62]	22,464,000,000 units/year	114,566,400 €/year
T7 RNA polymerase	38 €/mg	Merc ^i^	39 g/year	1,495,680 €/year
CleanCap Reagent (3’ OMe) AG	248 €/mg	Trilink Biotechnologies ^ii^	216 g/year	CleanCap Reagent (3’ OMe) AG
AFC resin	10,892 €/L (replacement every 100 cycles)	[60,61]	51 L/year	553,035 €/year
Ethanol	6.8 * €/kg	[58]	41,722 kg/year	283,707 €/year
Sodium citrate	0.7 * €/kg	[38]	102 kg/year	72 €/year
Dimethyldioctadecylammonium (DDAB)	554 €/g	Merc ^ii^^i^	8 kg/year	4,275,947 €/year
Cholesterol	11 €/g	Merc ^i^^v^	129,015 g/year	1,419,162 €/year
1,2-Dimyristoyl-*rac*-glycero-3-methoxypolyethylene glycol-2000 (DMG-PEG2000)	166 €/g	Merc ^v^	228,584 g/year	37,944,885 €/year
1,2-dioctadecanoyl-*sn*-glycero-3-phosphocholine (DSPC)	172 €/g	Merc ^v^^i^	65,996 g/year	11,351,268 €/year

^i^https://www.sigmaaldrich.com/catalog/search?term=rna+polymerase&interface=All&N=0&mode=partialmax&lang=en&region=GR&focus=product&page=2 (accessed on 27 August 2021); ^ii^ https://www.tebu-bio.com/?module=search2&brand=040 (accessed on 27 August 2021); ^iii^ https://www.sigmaaldrich.com/catalog/product/avanti/890810p?lang=en&region=GR&cm_sp=Insite-_-caSrpResults_srpRecs_srpModel_ddab-_-srpRecs3-3 (accessed on 27 August 2021); ^iv^ https://www.sigmaaldrich.com/catalog/product/sigma/c8667?lang=en&region=GR (accessed on 27 August 2021); ^v^ https://www.sigmaaldrich.com/catalog/product/avanti/880151p?lang=en&region=GR (accessed on 27 August 2021); ^vi^ https://www.sigmaaldrich.com/catalog/product/avanti/850365p?lang=en&region=GR (accessed on 27 August 2021); * Price after unit conversion.

## Data Availability

Not applicable.
